# Snail Shell-Reinforced Waste-Based Polymer Composites for Radiation Shielding and Anti-Reflective Applications

**DOI:** 10.3390/polym17233115

**Published:** 2025-11-24

**Authors:** Mustafa Ersin Pekdemir, Sibel Selçuk Pekdemir, Demet Yılmaz, Hatice Onay, Ibrahim Nazem Qader

**Affiliations:** 1 Polymer and Biomaterials Research Laboratory, Department of Chemistry, Faculty of Science, Firat University, 23119 Elazığ, Türkiye; ddemir@atauni.edu.tr; 2 Department of Physics, Faculty of Science, Atatürk University, 25240 Erzurum, Türkiye; inqader@gmail.com; 3 Department of Fishing Technology, Faculty of Fisheries, Recep Tayyip Erdogan University, 53100 Rize, Türkiye; hatice.bal@erdogan.edu.tr; 4 Department of Pharmacy, College of Pharmacy, Knowledge University, Erbil 44001, Iraq; ibrahimnazm@uor.edu.krd; 5 Department of Physics, College of Science, University of Raparin, Rania 46012, Iraq

**Keywords:** polyvinyl chloride (PVC), polystyrene blend, snail shell powder, radiation shielding composites, anti-reflective materials, waste-derived filler

## Abstract

The increasing demand for sustainable and multifunctional materials in radiation shielding and optical applications has driven research toward utilizing natural and waste-derived reinforcements in polymer matrices. However, achieving effective attenuation performance across different radiation types using eco-friendly fillers remains a significant challenge. In this study, polyvinyl chloride (PVC)/Polystyrene (PSt) blend composites (1:1 weight ratio) were reinforced with powdered snail shell (SSP) as a biogenic additive, aiming to enhance their shielding and optical performance. Composites containing 5%, 10%, 20%, and 30% SSP (*w*/*v*) were fabricated and characterized. Key parameters including linear attenuation coefficient (LAC), mass attenuation coefficient (MAC), mean free path (MFP), half-value layer (HVL), and effective atomic number (Zeff) were measured using a variable-energy X-ray source (13.37–59.54 keV) and ULEGe detector. Fast neutron shielding performance and theoretical values for build-up factor (EBF) and macroscopic neutron cross-sections were also calculated. The results showed a marked improvement in X-ray attenuation with increasing SSP content (SSP30 > SSP20 > SSP10 > SSP5), while neutron shielding declined due to the high oxygen content of SSP. Among the tested samples, the SSP30 composite exhibited the highest X-ray attenuation efficiency, whereas the SSP5 composition showed the greatest enhancement in optical reflectance and neutron absorption, indicating optimal performance in these respective tests. Additionally, 5% SSP incorporation improved optical reflectance by 12%, indicating enhanced photon backscattering at the material surface. This behavior contributes to improved gamma shielding efficiency by reducing photon penetration and enhancing surface-level attenuation. These findings highlight the potential of snail shell-based fillers as low-cost, sustainable reinforcements in multifunctional polymer composites.

## 1. Introduction

Polyvinyl chloride (PVC) and polystyrene (PSt) are two of the most utilized thermoplastics in commercial and industrial sectors due to their affordability, processability, and chemical resistance. However, their environmental impact, particularly in the form of persistent waste, has driven the search for more sustainable material solutions. One emerging approach is the development of polymer blends and composites from recycled plastics and natural fillers. In fact, polymer blending offers a versatile “green” strategy to tailor polymer properties without the need for entirely new synthesis, reducing cost and accelerating development of functional materials. PVC/PSt blends are especially promising, as they allow for the adjustment of mechanical and thermal properties through a simple and cost-effective method. In parallel, the use of biogenic waste such as snail shells—a calcium carbonate-rich byproduct from the food and agriculture industries—has gained increasing attention [1]. These discarded shells, once considered waste, are now being explored as valuable resources for reinforcing polymer matrices due to their mineral content, structural integrity, and broad functional potential across industries including construction, filtration, cosmetics, and biomedicine [2]. Such strategies align with the broader trend of utilizing natural and industrial wastes to develop sustainable and renewable polymer composites [3].

Despite the widespread use of synthetic polymers, their inherent drawbacks—such as brittleness, limited thermal resistance, and low biodegradability—pose challenges for long-term performance and environmental sustainability. To address these issues, researchers have turned to modification strategies like polymer blending and the incorporation of natural or inorganic fillers. Among these, polymer blending offers significant advantages over chemical copolymerization or the synthesis of new monomers. It is more adaptable, scalable, and cost-efficient, allowing for property tuning through careful selection of blend ratios and fillers [4,5,6]. Additionally, using biowaste-derived materials like snail shells helps reduce raw material costs, lower environmental impact, and enhance performance characteristics, especially when supported by methods that ensure good dispersion and interaction within the polymer matrix [7,8,9]. Recent studies have also demonstrated the effectiveness of one-step fabrication techniques in improving the functional properties of polymer-based membranes, underscoring the practical value of such simple and scalable approaches for composite development [10]. This approach is also more practical than developing new monomers, which is typically time-consuming and expensive [11,12]. Additionally, incorporating fillers such as bio-waste or inorganic particles helps enhance thermal resistance, mechanical strength, and barrier properties, especially when supported by proper dispersion techniques [13]. Similar improvements in composite properties have been reported for synthetic fillers such as carbon black in polymer blends like PA6/HDPE, highlighting the versatility of filler-based reinforcement strategies across diverse systems [14].

Compared to other bio-fillers such as rice husk, coconut shell, or eggshell powder, snail shell particles (SSPs) offer a unique combination of high calcium carbonate purity, naturally occurring hydroxyapatite, and favorable particle morphology, all of which contribute to superior interfacial bonding and dispersion within composite matrices. Notably, snail shells from various species exhibit exceptionally high CaCO_3_ content—for example, Helix pomatia shells contain approximately 97% CaCO_3_, Cornu aspersum shells contain 95–99%, and Eobania vermiculata shells are reported to be nearly 100% CaCO_3_ [15]. These intrinsic properties enable SSPs to outperform other natural fillers in enhancing mechanical strength, thermal stability, and environmental resistance in composite applications.

Several studies have explored the incorporation of SSPs as a sustainable and cost-effective reinforcement material in polymer and metal matrix composites [16]. These shells, rich in calcium carbonate and hydroxyapatite, offer excellent mechanical and thermal properties when processed appropriately [17]. Syamimi et al. [18] demonstrated that adding SSPs to epoxy resin significantly enhanced both tensile and flexural strength, particularly at 10–15 wt.% loading, with additional improvements observed when heat-treated SSPs were used. Their results also indicated an increase in thermal stability and glass transition temperature of the composites. Similarly, Oladele et al. [19] found that bio-derived hydroxyapatite from snail shells improved the mechanical, wear, and thermal properties of epoxy composites, with optimal performance at 15 wt.% reinforcement. Gbadeyan et al. [20] investigated epoxy composites reinforced with snail and eggshell fillers, reporting substantial improvements in stiffness, impact resistance, and water resistance—especially in hybrid systems—due to the high calcium carbonate content. In a study by Udoye et al. [21], SSPs were successfully used to reinforce aluminum matrix composites, where uniform particle dispersion enhanced electrical conductivity and structural integrity. Karaoui et al. [22] further confirmed the potential of SSPs in polymer matrices by incorporating them into PSt. They reported increased modulus, hardness, and thermal resistance, particularly at 10 wt.% SSP loading. Additionally, recent works employing seashell or shell-derived CaCO_3_ fillers in epoxy or bio-resin systems demonstrate improved thermal and mechanical performance, highlighting the broad applicability of biogenic fillers [23]. Although prior studies have employed snail shell (or general shell-derived CaCO_3_) as bio-fillers in polymer composites (especially thermosets) [24], the use of SSPs in a recycled PVC/PSt blend for the dual goals of radiation shielding and anti-reflective functionality remains unexplored. Previous works have largely focused on mechanical, wear, or thermal enhancements in thermoset systems, rather than multifunctional shielding and optical performance in thermoplastic blends. Moreover, in radiation-shielding polymer composites the emphasis has been mostly on inclusion of heavy metal oxides or high-Z fillers (e.g., Bi, W, Pb-based fillers) to improve X-ray/gamma attenuation [25], rather than employing waste-derived, low-cost biogenic fillers that can simultaneously influence optical reflectance behavior. Therefore, this study aims to expand that scope by evaluating SSP-filled composites under broader performance metrics, positioning SSPs as a multifunctional, bio-derived alternative for advanced composite applications.

This study aims to synthesize and characterize PVC/PSt-based nanocomposite films reinforced with SSPs, utilizing both recycled polymers and biogenic waste. Waste PVC and PSt foam were first purified and then blended in equal weight ratios using tetrahydrofuran (THF) as the solvent. SSPs was added in varying concentrations—5%, 10%, 20%, and 30% by weight of the total polymer content—yielding the samples SSP5, SSP10, SSP20, and SSP30. The films were fabricated via solution casting and dried under controlled conditions. The resulting materials were subjected to structural, morphological, and thermal analyses to investigate how SSP content affects the properties of the composites. This research contributes to the growing field of sustainable material science by turning waste into valuable resources for enhanced, eco-friendly polymer systems.

## 2. Materials and Methods

### 2.1. Materials

Waste PVC was supplied by Akkim Kimya (Yalova, Türkiye) and was used after purification. Tetrahydrofuran (THF) (≥99.9%, anhydrous, inhibitor-free), used to dissolve the polymers, was purchased from Merck (Darmstadt, Germany).

### 2.2. Preparation of Snail Shell Powder

Specimens of Rapana venosa were collected from the southeastern coast of the Black Sea, specifically from the shores of Rize, Turkey (41°01′42.0″ N, 40°32′00.8″ E), in December 2024. Prior to powder preparation, the shells underwent a thorough cleaning process to remove residual soft tissues, sand, and other debris. They were soaked in distilled water for 48 h and subsequently rinsed multiple times to ensure complete cleanliness. The cleaned shells were then dried in an oven at 60 °C. Once dried, the shells were initially broken into smaller fragments using a mortar and pestle, followed by fine grinding into powder using a high-speed blender (Waring, Model HGB2WTS3, Torrington, CT, USA).

### 2.3. Purification of Box Filling Foam

The box filling foam used in this study was first subjected to a purification process. A known quantity of the foam was weighed and dissolved in tetrahydrofuran (THF) under continuous stirring using a magnetic stirrer. The resulting solution was then added dropwise into ethyl alcohol while stirring vigorously to induce precipitation. The precipitated PSt was collected by filtration using filter paper and subsequently dried in an oven at 40 °C under ambient conditions.

### 2.4. Preparation of PVC/PSt Blend Films

Polymer blend films were prepared by combining purified PSt and PVC in a 1:1 weight ratio. Specifically, 0.25 g of PVC was dissolved in 5 mL of THF in a sealed container under continuous stirring. Once fully dissolved, 0.25 g of the previously purified PSt was added to the solution and stirring was continued until a homogeneous, transparent blend was obtained. The resulting solution was carefully poured into a Petri dish and dried in an oven at 40 °C.

### 2.5. Preparation of PVC/PSt/SSP Nanocomposite Films

**Figure 1:**
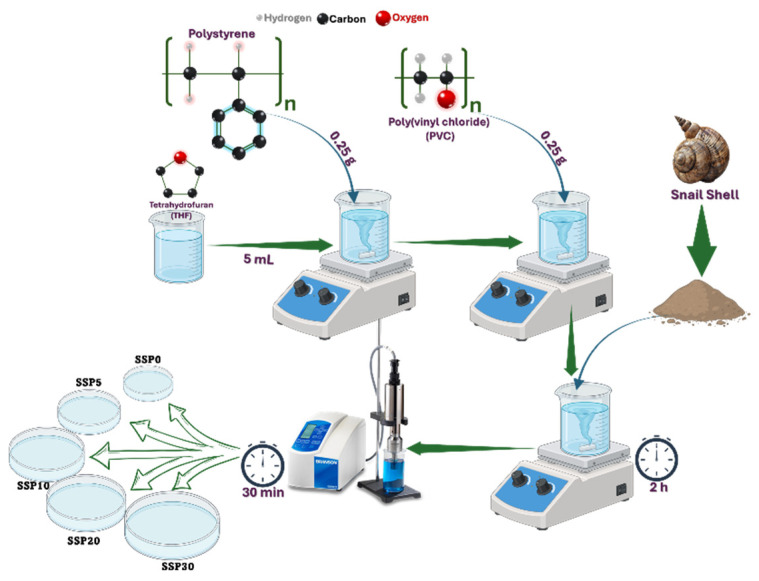
Preparation of PVC/PSt/SSP composite films.

PVC/PSt/SSP nanocomposite films were fabricated by incorporating SSPs into the PVC/PSt matrix. The SSP was added in varying concentrations of 5%, 10%, 20%, and 30% by weight relative to the total polymer content. The prepared samples were labeled as SSP0 (control), SSP5, SSP10, SSP20, and SSP30, respectively. For each composite, 0.025 g of SSP was added to the pre-prepared homogeneous PVC/PSt solution. The mixture was stirred for 2 h using a magnetic stirrer, followed by dispersion in an ultrasonic homogenizer for 30 min to ensure uniform nanoparticle distribution. The final mixtures were cast into Petri dishes and dried in a vacuum oven at 40 °C. The preparation process is illustrated in Figure 1, while the sample codes, weight fractions (%), and densities of the PVC/PSt/SSP composites are presented in Table 1.

### 2.6. High-Energy Experimental Procedure

#### 2.6.1. Photon Attenuation Measurement Procedure

**Figure 2:**
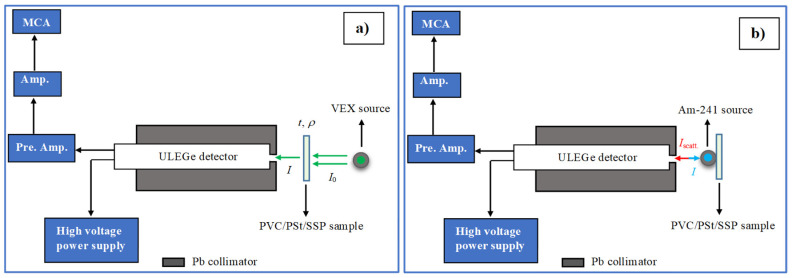
(**a**) Absorption geometry, (**b**) Backscattering geometry in gamma spectroscopy.

Figure 2a illustrates the experimental setup used for photon attenuation measurements, which consists of a variable energy X-ray (VEX) source, the sample holder, and a semiconductor detector. The VEX source is capable of producing X-rays of varying energies, depending on the choice of secondary target material. These secondary targets were irradiated with 59.54 keV gamma photons emitted by a 3 × 10^7^ Bq Am-241 source, resulting in the emission of characteristic K X-rays.

In this study, secondary targets made of copper (Cu), rubidium (Rb), molybdenum (Mo), silver (Ag), barium (Ba), and terbium (Tb) were employed to generate different photon energies. The characteristic K X-rays from selected secondary targets (Rb, Mo, Ag, and Tb) were directed toward the PVC/PSt/SSP nanocomposite samples. The specific photon energies produced by the VEX source for each target material are listed in Table 2.

**Figure 3:**
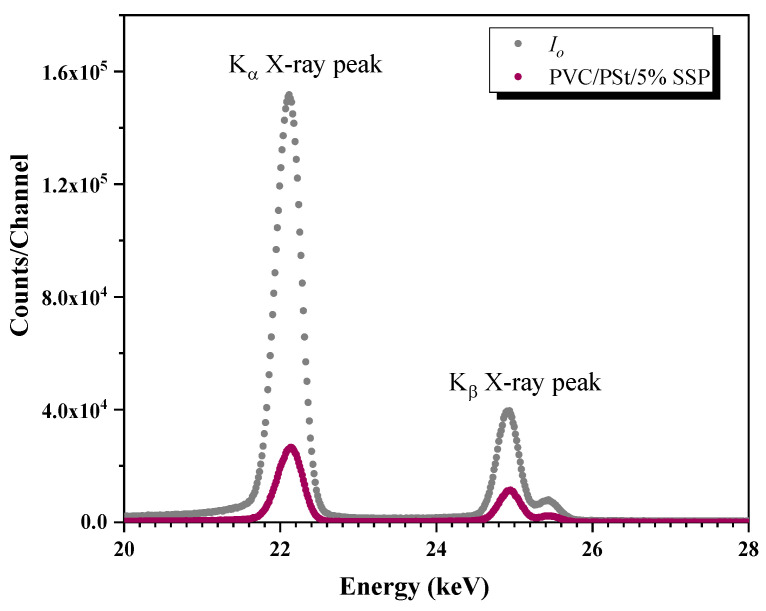
The transmission spectra of PVC/PSt/5% SSP for Ag K X-rays from VEX source.

Photon intensities were measured both before and after passing through the samples using a ”ULEGe (Ultra-Low Energy Germanium) (Mirion Technologies, Torrington, CT, USA) detector. The gamma spectra of each sample were recorded over a period of 3600 s to ensure statistical reliability. A representative transmission spectrum for the PVC/PSt/SSP5 sample is shown in Figure 3.

To determine the photon attenuation parameters, the Lambert–Beer law was applied [26]:

(1) $$I=I_{o}e^{-\mu x}$$

where $I_{o}$ and are the incident and transmitted photon intensities, respectively, *µ* is the linear attenuation coefficient, and *x* is the sample thickness. From this relationship, key shielding parameters such as the linear attenuation coefficient (LAC), mass attenuation coefficient (MAC), mean free path (MFP), and half-value layer (HVL) were calculated for each sample.

#### 2.6.2. Albedo Number Determination

The experimental setup for determining the albedo number (backscattering probability) is shown in Figure 2b. In this setup, the PVC/PSt/SSP samples were irradiated with 59.54 keV gamma photons emitted from an Am-241 radioactive source. The backscattered photons were detected at a scattering angle of 180° using a ULEGe detector, allowing for the analysis of photons that were reflected directly backward from the sample surface. The energy spectra obtained from the detector were analyzed to distinguish between coherent (Rayleigh) and incoherent (Compton) scattering peaks. Data acquisition was carried out for 7200 s for each sample to ensure high-resolution and reliable spectral information. The methodology for calculating albedo parameters, including the extraction of peak intensities and relevant scattering information, follows standard procedures detailed in the literature [27].

#### 2.6.3. Neutron Attenuation

**Figure 4:**
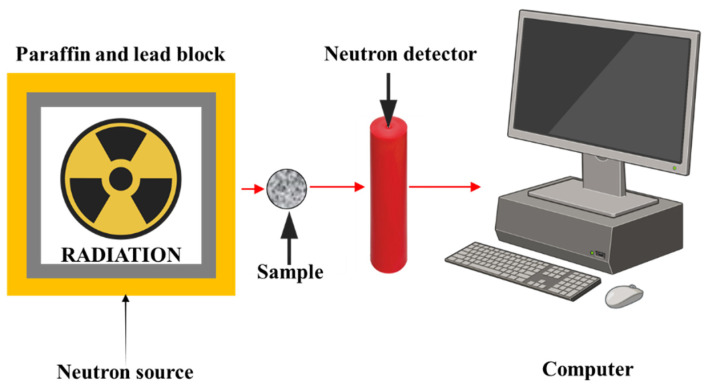
The experimental geometry for neutron dose measurements.

To assess the neutron shielding capability of the composite samples, fast neutron equivalent dose rates were measured using a BF_3_ (boron trifluoride) proportional counter. The measurements were performed with a standard Am-Be (Americium–Beryllium) neutron source, which emits fast neutrons in a broad energy spectrum. The experimental arrangement for neutron measurements is illustrated in Figure 4. The absorbed neutron dose equivalent was determined for each PVC/PSt/SSP sample. In addition, the fast neutron removal cross-section (ΣR), thermal and fast neutron macroscopic cross-sections, and other relevant neutron interaction parameters were theoretically calculated to support the experimental findings. All physical models, formulas, and computational methods used in the determination of neutron shielding parameters are summarized in Table 3.

### 2.7. Characterization Methods

Attenuated total reflection infrared (ATR-IR) spectra obtained for functional group analysis of SSP-doped PVC/PSt composite films were performed on a Diamon/ZnSe crystal plate (PerkinElmer Inc., Waltham, MA, USA) in the 4000–550 cm^−1^ wavenumber range. Thermal stability studies and calorimetric measurements of the composite films were performed simultaneously with the Hitachi Simultaneous Thermogravimetric Analyzer (STA) (Hitachi High-Tech Science Co., Ltd., Tokyo, Japan) device under N_2_ gas with a heating rate of 10 °C/min. The structure and morphology of PVC/PSt blend film and SSP-doped composite films were examined by SEM (Scanning Electron Microscopy)/EDX (Energy Dispersive X-ray Spectroscopy) (LEO Evo-40 VPX, LEO Electron Microscopy Ltd., Cambridge, UK). The XRD patterns of composite films were examined with a compact bench-top XRD (Rigaku miniflex 600, Rigaku Corporation, Tokyo, Japan) in the 2θ range of 10–80°.

## 3. Results and Discussions

### 3.1. ATR-IR Analysis

**Figure 5:**
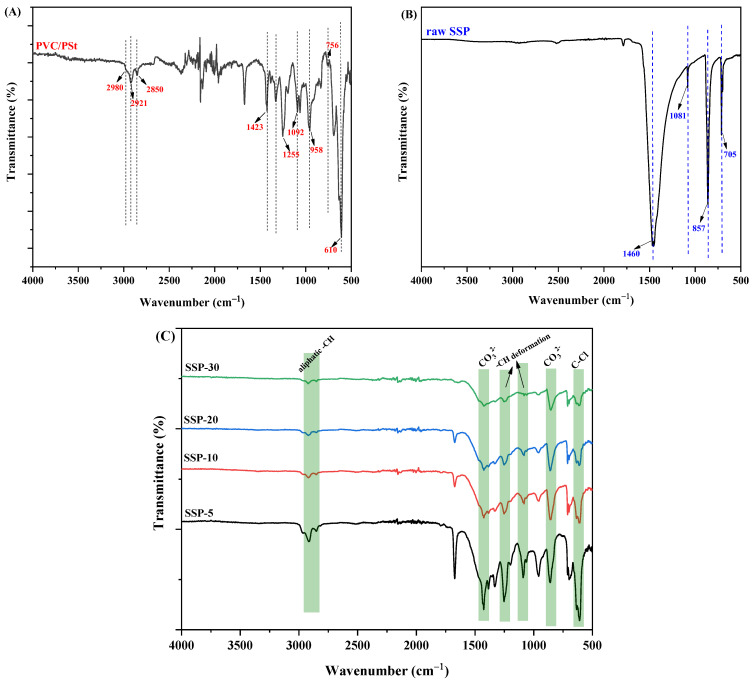
ATR-IR spectrum of (**A**) PVC/PSt blend, (**B**) raw SSP and (**C**) PVC/PSt/SSP composite films.

The ATR-IR spectra of the pure PVC/PSt blend film and the composite films incorporating SSPs are presented in Figure 5. In the spectrum of the pure polymer blend (Figure 5A), the peak at 2980 cm^−1^ is attributed to the aromatic –CH stretching of PSt. The fork-shaped signals observed at 2921 cm^−1^ and 2850 cm^−1^ correspond to the asymmetric and symmetric stretching vibrations of –CH_3_ and –CH_2_ groups, present in both PVC and PSt [31]. The peak at 1423 cm^−1^ is characteristic of the methylene group wagging vibration in PVC [32]. An overlapping signal around 1255 cm^−1^ arises from both the aromatic C–H deformation of PSt and the –CH bending vibrations adjacent to chlorine atoms in PVC [9]. Additional distinctive bands include the aromatic –CH deformation of PSt at 1092 cm^−1^, –CH_2_ rocking vibrations in the 958–756 cm^−1^ range, and the C–Cl stretching of PVC at 610 cm^−1^ [33,34].

Figure 5B displays the ATR-IR spectrum of raw SSP, which shows distinct signals associated with the carbonate ion (CO_3_^2−^). These include characteristic peaks at 1460 cm^−1^, 1081 cm^−1^, 857 cm^−1^, and 705 cm^−1^, attributed to the asymmetric stretching, out-of-plane bending, and in-plane bending modes of the calcite C–O bond [35,36,37].

In Figure 5C, the spectra of the PVC/PSt/SSP composite films are shown. The presence of all the aforementioned characteristic bands from both the polymer blend and SSP confirms successful incorporation of the filler into the matrix. Notably, a progressive decrease in the intensity of certain polymer-specific bands—such as the C–Cl stretching at 610 cm^−1^, the aromatic –CH deformation between 1095 and 1255 cm^−1^, and the CH stretching bands in the 2980–2850 cm^−1^ region—is observed with increasing SSP content. This attenuation is consistent with increasing filler loading and possible partial masking or scattering of polymer vibrations by SSP particles. Importantly, no new absorption bands or definitive peak shifts beyond resolution are observed, which suggests that no strong covalent bond is formed between the polymer chains and the filler. Instead, the spectral changes are likely due to physical interactions such as van der Waals forces, hydrogen bonding via surface –OH on the snail shell, or interfacial adhesion (mechanical interlocking/physical embedding). This interpretation aligns with prior studies on PVC/CaCO_3_ systems, where FTIR changes are limited to intensity variations without novel band emergence, reflecting compatibility rather than chemical reaction [38].

### 3.2. Thermal Analysis (TGA and DSC)

**Figure 6:**
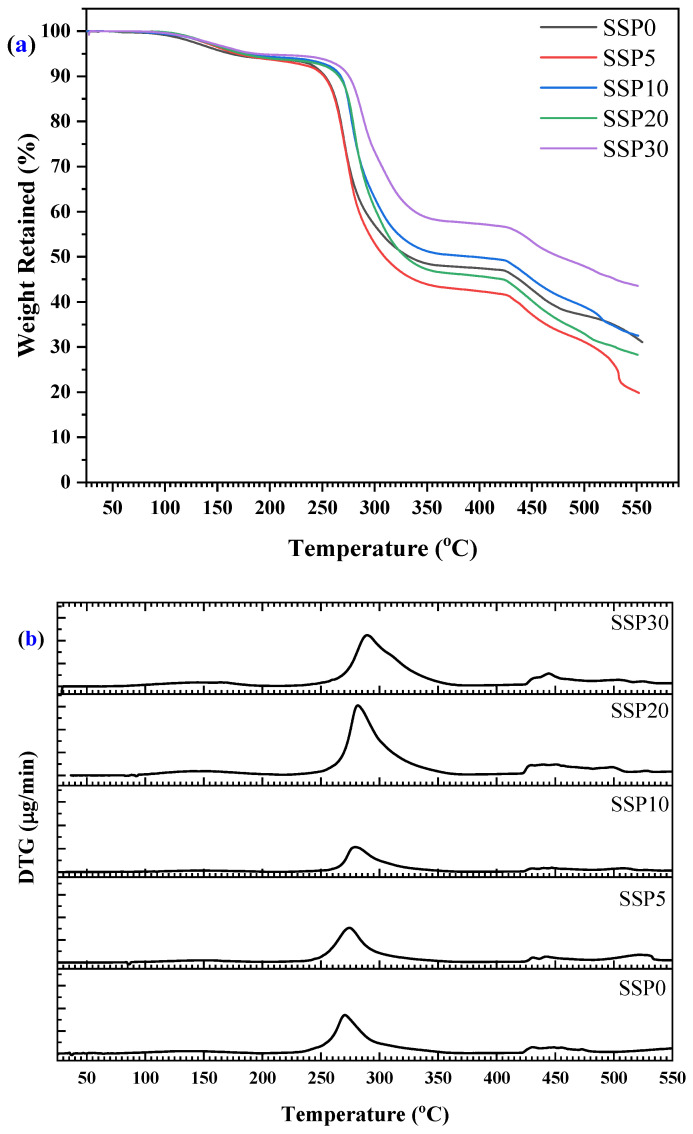
(**a**) TGA and (**b**) DTG curves of PVC/PSt/SSP composite films.

Based on the thermogravimetric analysis (TGA) results presented in Figure 6a (TGA curve) and Figure 6b (DTG curve), the thermal behavior of the SSP-based composite films revealed a four-step degradation process occurring between 20 °C and 550 °C. The first stage of weight loss, observed around 100 °C, is relatively minor and can be attributed to the evaporation of adsorbed moisture [39] and residual solvents from the film surface. This initial loss is common in polymer composites and does not indicate any chemical decomposition of the polymer matrix or filler. The second major degradation step, centered at approximately 265 °C, likely corresponds to the initial breakdown of the polymer chains [40] in the PVC/PSt blend. PVC, in particular, is known to undergo dehydrochlorination beginning around 220–390 °C [41], releasing hydrogen chloride (HCl) and leading to the formation of conjugated polyene sequences. This breakdown accelerates thermal degradation and alters the mechanical and optical properties of the polymer [42]. The third and most significant mass loss stage occurs around 425 °C, indicating the decomposition of both the remaining polymer backbone and organic components within the SSPs, primarily proteins and other biogenic organics. On the other hand, PSt undergoes a single-phase degradation between 390 and 480 °C, which corresponds to the fourth step of the decomposition process [43].

Interestingly, the degradation process continues even after 500 °C without reaching a clear plateau, suggesting that complete decomposition of the organic content has not yet occurred. Since the system contains only organic and biogenic materials, the absence of a plateau indicates the ongoing degradation and volatilization of complex molecules. Unlike systems reinforced with purely inorganic fillers, the SSPs used here is composed largely of calcium carbonate (CaCO_3_) [44], along with minor quantities of organic matrix components. However, the presence of CaCO_3_ typically contributes to higher thermal stability at elevated temperatures due to its decomposition into calcium oxide (CaO) and carbon dioxide (CO_2_) [45] above 700 °C—a behavior not fully captured within the temperature range of this study. Such barrier effects of inorganic fillers are well known in polymer composites, where fillers can slow down diffusion of volatile products or reduce heat conduction.

These results suggest that while SSP contributes to a composite structure that degrades in multiple stages, it still provides a level of thermal resistance useful for medium-temperature applications. The absence of a stable residue at the end of the test reinforces the conclusion that the composites are almost entirely organic and susceptible to complete thermal degradation. With further heating, one might observe the typical CaCO_3_ to CaO transformation known for snail shells, which is of interest for applications requiring thermal shielding or the development of char-forming composites.

**Figure 7:**
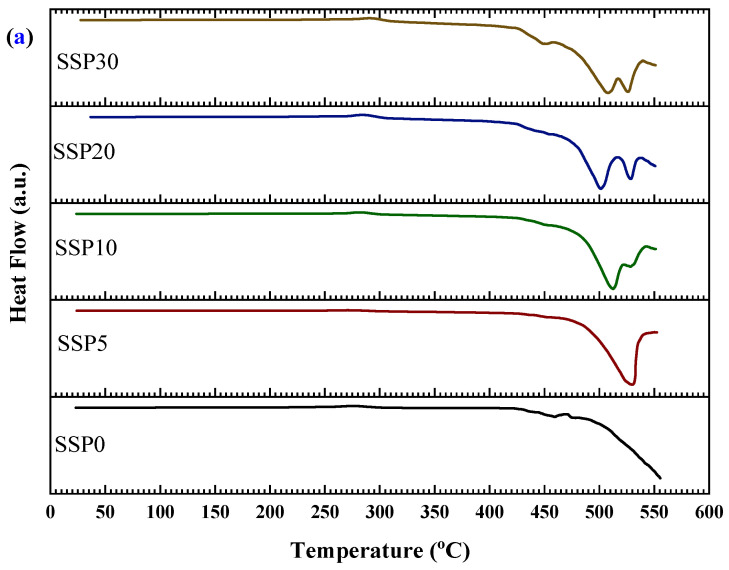

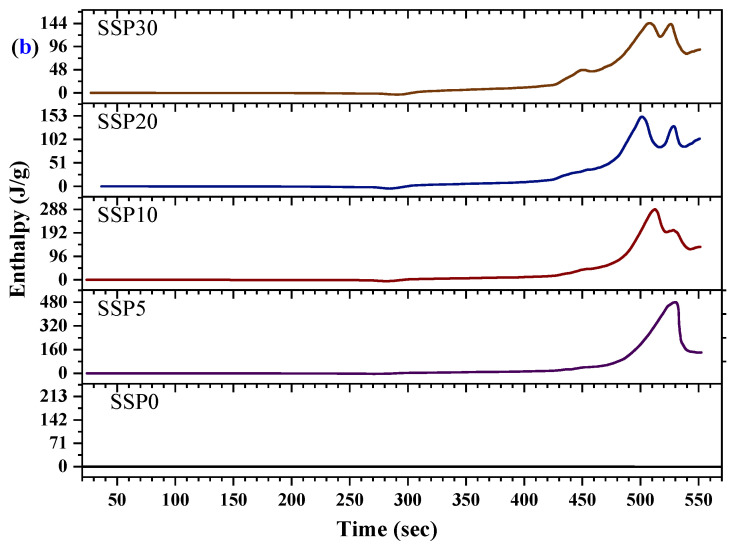
(**a**) DSC curves and (**b**) enthalpy changes in PVC/PSt/SSP composite films.

The thermal transitions of the PVC/PSt/SSP composite films were further investigated using DSC, as presented in Figure 7a, with the corresponding enthalpy changes shown in Figure 7b. All samples exhibited pronounced endothermic peaks, indicating elevated-temperature thermal processes such as structural relaxations, molecular rearrangements, and decomposition of polymer segments or residual organic filler components. For the control sample (SSP0), which contains no snail shell powder, the DSC curve revealed a strong endothermic event starting around 480 °C, which we now interpret as the onset of decomposition of the PVC/PSt matrix under the applied heating rate, rather than as a fusion process. Upon incorporation of 5% SSP into the blend (sample SSP5), the thermal profile shifts: a broad endothermic peak begins near 465 °C and extends toward the instrument’s upper temperature limit. This shift suggests that the presence of SSP slightly modifies the thermal behavior of the matrix, perhaps by affecting heat transfer, delaying volatile release, or altering decomposition kinetics. As the SSP content increases to 10%, 20%, and 30%, the DSC curves display broader and sometimes split endothermic events, indicative of multiple overlapping decomposition pathways from both polymer components and biogenic residues in SSP. A subtle shoulder in the ~400–450 °C range—more visible in composites with higher SSP loading—may correspond to early-stage breakdown of residual organic materials in the shell. The evolution of these DSC profiles with increasing SSP content reflects increased complexity of thermal degradation behavior in the composites. The enthalpy data in Figure 7b, showing increasing energy absorption with higher SSP loading, are now understood as evidence of more demanding decomposition mechanisms and interfacial complexity, rather than fusion enthalpies.

Moreover, the shift in thermal transitions and the increased complexity of the DSC profiles with SSP addition points toward enhanced thermal interactions between the filler and the polymer matrix. The SSPs appear to act as a structural modifier, influencing the degradation temperature of the composite. This is consistent with earlier TGA observations that the thermal degradation continues beyond 500 °C without stabilizing, further supporting the idea that SSP introduces multi-phase degradation behavior. These changes suggest improved thermal resistance and a modified degradation mechanism, making the SSP-based composites promising candidates for thermally stable biopolymer applications.

### 3.3. Surface Morphology

**Figure 8:**
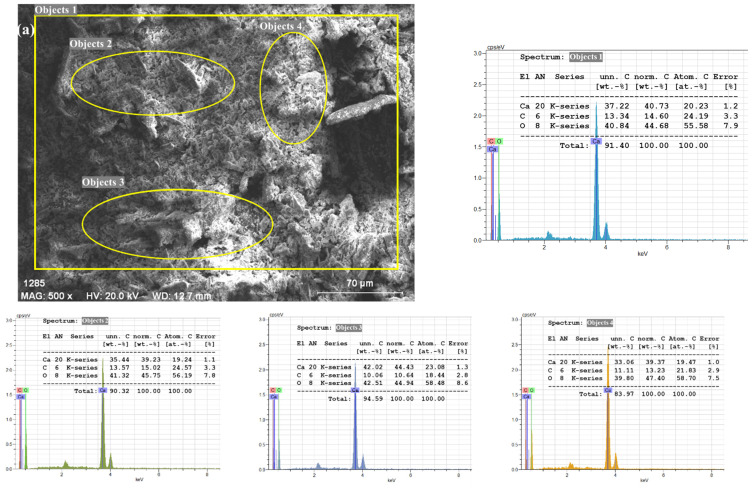

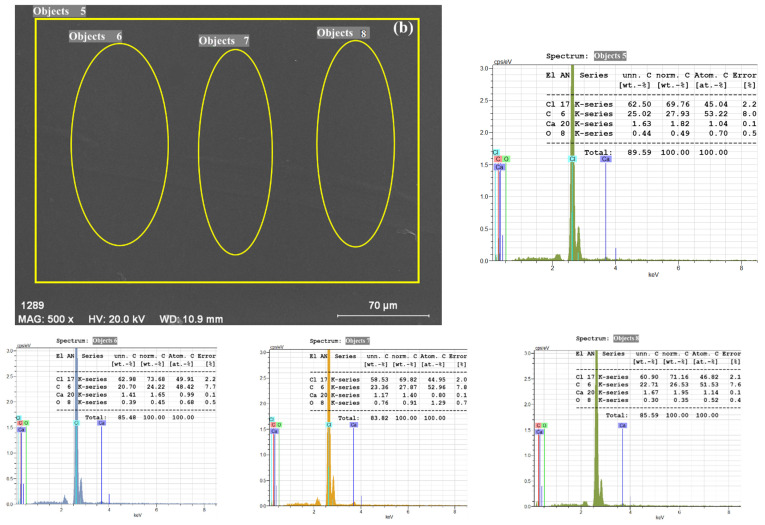
SEM/EDX images of PVC/PSt/SSP composite films; (**a**) SSP, (**b**) SSP30.

The surface morphology and elemental composition of the SSPs and the PVC/PSt/SSP nanocomposite films were analyzed using SEM coupled with EDX. Figure 8a presents the SEM micrograph and corresponding EDX spectrum of the neat SSPs. The surface morphology of the SSP shows irregularly shaped particles with varying sizes, typical of naturally derived biogenic calcium carbonate. The powder particles exhibit a relatively rough texture and appear as aggregates with micro- and nanoscale dimensions, which is advantageous for enhancing the interaction between the filler and polymer matrix. The EDX spectrum obtained from the SSP confirms the elemental composition of the snail shell, which predominantly includes calcium (Ca), carbon (C), and oxygen (O) [46]. These elements are characteristic of calcium carbonate (CaCO_3_), which exists in nature in several polymorphic forms, including calcite, aragonite, and vaterite. The strong Ca peak, along with the well-defined C and O peaks, further supports the presence of crystalline CaCO_3_ as the main component. The absence of other impurity peaks also indicates that the washing, drying, and grinding processes employed in preparing the SSP were effective in producing a relatively pure biogenic filler suitable for composite applications.

In addition, the EDX quantitative table shown alongside the spectrum confirms that Ca, C, and O constitute more than 95 wt.% of the total composition, with only trace amounts of other elements, further validating the purity of the biogenic CaCO_3_ filler. The inclusion of both the SEM image and EDX spectrum in the same panel allows direct correlation between particle morphology and elemental composition, confirming the reliability of the analysis.

Figure 8b illustrates the surface morphology of the SSP30 composite, containing 30 wt.% SSPs in a 1:1 PVC/PSt matrix, captured at a magnification corresponding to a 70 µm scale bar. At this scale, the surface appears smooth and homogeneous, with no visible agglomerates or exposed filler particles, suggesting excellent dispersion and integration of CaCO_3_ within the polymer blend. The circled area and points labeled 5–7 indicate regions selected for EDX analysis, aimed at verifying elemental distribution across different zones. These markers do not correspond to a visible filler but serve to confirm the even incorporation of SSP within the composite matrix.

The EDX spectrum of the SSP30 film, obtained from a large-area scan as well as three different localized regions, consistently reveals peaks corresponding to the elements present in the polymer blend and the filler. The dominant peaks in the spectrum are attributed to chlorine (Cl), carbon (C), and oxygen (O), consistent with the elemental composition of PVC and PSt. A relatively small but distinct calcium (Ca) peak is also observed in all three regions. The presence of Cl is indicative of the PVC component [47], while C and O originate from both the organic polymeric matrix and the CaCO_3_ filler. The weak but noticeable Ca peak across all examined regions confirms the successful and uniform incorporation of CaCO_3_ nanoparticles into the blend matrix, even at high filler loadings.

Notably, the lack of localized regions with significantly higher Ca signals or visible clusters in the SEM image supports the conclusion that the SSP particles were well distributed throughout the polymer network. This uniform dispersion is critical for achieving consistent material performance, as it minimizes stress concentration points and enhances mechanical and thermal properties.

### 3.4. XRD Analysis

**Figure 9:**
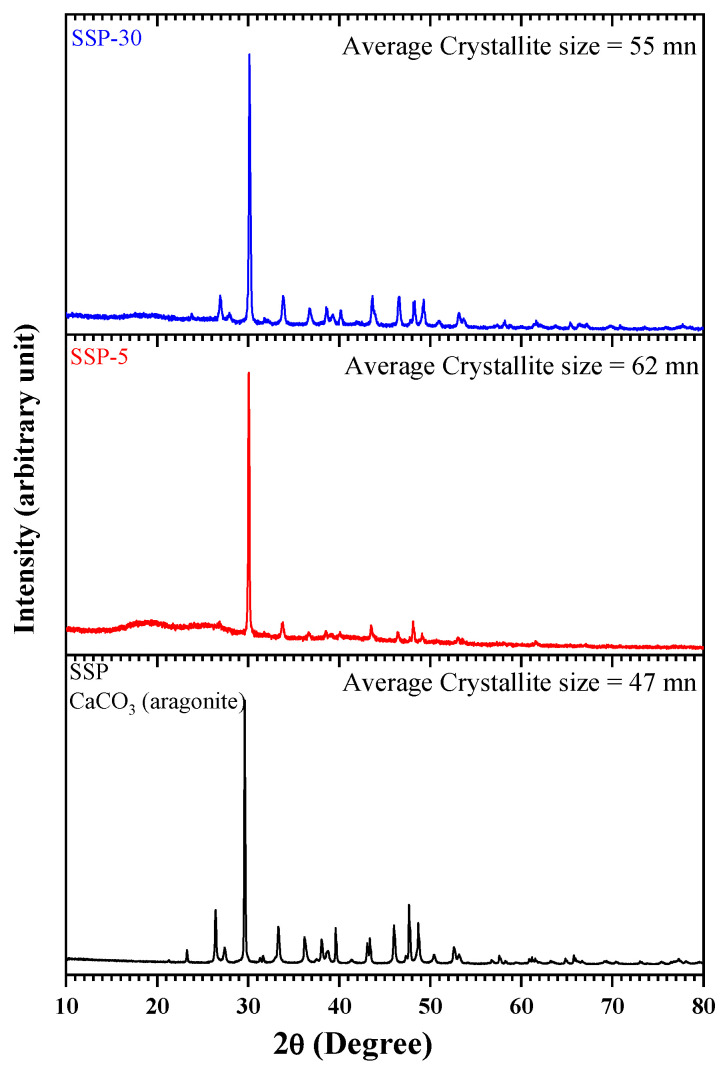
XRD patterns of PVC/PSt/SSP composite films.

The X-ray pattern given in Figure 9 provided information about the crystal structure of SSPs and composite films prepared with polymer blend. When the pattern of pure SSP was examined, it was seen that the distinct peaks belonged to the aragonite form of CaCO_3_ with orthorhombic structure. Aragonite form represents the metastable polyform of CaCO_3_ at room conditions and atmospheric pressure [37,48]. Characteristic major peaks indicating the presence of CaCO_3_ in aragonite crystal form appeared at 23.34°, 29.63°, 36.20°, 39.67°, 43.36°, 47.65°, 48.69°, 57.54°, 61.17° and 65.83°. The reflections corresponding to these sharp signals are (012), (104), (110), (113), (202), (024), (116), (122), (119) and (002), respectively. The international data diffraction center says that these data belong to the Aragonite crystal with the orthorhombic form of CaCO_3_ [49]. In the XRD spectra of the composite films with a PVC/PSt blend and SSP content (5% and 30%), the intensity of the characteristic SSP peaks increased with increasing SSP content. This can be interpreted as evidence of the increase in SSP content in the composite.

X-ray diffraction (XRD) analysis was performed to investigate the crystalline structure of the SSPs and the PVC/PSt/SSP composite films. The XRD patterns revealed characteristic diffraction peaks corresponding to the crystalline phases of calcium carbonate (CaCO_3_), the principal inorganic component of snail shells. The degree of peak broadening was analyzed to estimate the average crystallite size of the samples using the Scherrer Equation (2):

(2) $$D=\frac{K\;\lambda }{\beta \cos\theta }\;,$$

where *D* is the average crystallite size (nm), *K* is the shape factor (typically 0.9), *λ* is the wavelength of the X-ray source (1.5406 Å for Cu Kα radiation), *β* is the full width at half maximum (FWHM) of the diffraction peak (in radians), *θ* is the Bragg angle (in degrees). The calculated crystallite sizes were 47 nm for neat SSP, 62 nm for SSP-5 (5 wt.% SSP), and 55 nm for SSP-30 (30 wt.% SSP). These crystallite sizes refer to coherent crystalline domains and are not directly equal to the physical particle sizes, which would require techniques such as TEM to measure. The initial increase in crystallite size observed in the SSP-5 sample suggests that low filler content may promote slight aggregation or reorganization of crystalline domains within the polymer matrix. However, the subsequent reduction in crystallite size for SSP-10 indicates that higher SSP content leads to improved dispersion of the filler particles, which can limit crystal growth or introduce lattice distortions due to increased polymer–filler interaction. These results suggest that the addition of SSPs significantly affects the crystallite size and, by extension, the structural characteristics of the composite films. Optimizing the filler content is therefore essential to achieving desired crystallinity, which influences key material properties such as mechanical strength, thermal stability, and barrier performance. Further analysis will be necessary to correlate these structural findings with functional properties of the developed nanocomposites.

### 3.5. High Energy Experiments

#### 3.5.1. Photon Attenuation

The experimentally determined mass attenuation coefficients (MAC) of PVC/PSt composites containing SSPs were compared with theoretical values calculated using the EpiXS software, as summarized in Table 4. A strong agreement was observed between the experimental and theoretical results, with discrepancies ranging from approximately 1.95% to 5.79% across the energy spectrum of 13.37–59.54 keV. The experimental uncertainty remained below 5.8%.

**Figure 10:**
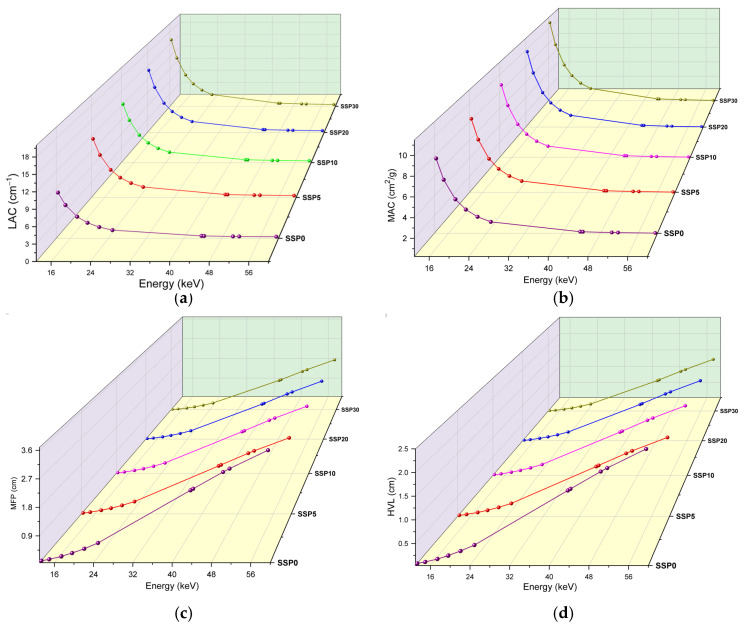
(**a**) LAC values, (**b**) MAC values, (**c**) MFP values, and (**d**) HVL values of PVC/PSt/SSP composite films.

The variations in the linear attenuation coefficient (LAC) and MAC as a function of photon energy are illustrated in Figure 10a and Figure 10b, respectively. These coefficients are key parameters that characterize a material’s ability to attenuate X-rays or gamma rays. Both LAC and MAC are influenced by the incident photon energy and the atomic composition of the absorber. As expected, both coefficients generally decrease with increasing photon energy due to changes in the dominant photon–matter interaction mechanisms.

At low photon energies, MAC values decrease sharply with energy, typically following a power law behavior proportional to *E*^−3^ to *E*^−4^. This results in strong attenuation at low energies, where the photoelectric effect is dominant. In the intermediate energy region, Compton scattering becomes the principal interaction mechanism, leading to a more gradual decrease in MAC. At higher energies (≥1.022 MeV), pair production may become significant, especially for high atomic number (Z) materials, potentially causing the MAC to rise again. Specifically, in this study, an average drop in MAC values to around 0.44 cm^2^/g was observed after 24.9 keV (see Figure 10b).

The inclusion of SSP in the polymer matrix significantly enhanced the attenuation performance. At 13.37 keV, the absorption coefficients increased by approximately 5.1% and 38.7% for SSP10 and SSP30 samples, respectively, compared to the unfilled polymer blend. The effectiveness of photon attenuation among the samples follows the order: SSP30 > SSP20 > SSP10 > SSP5. Among them, the SSP30 sample demonstrated the highest MAC values, indicating superior gamma-ray shielding capability and highlighting its potential for radiation protection applications. Also, using the experimental MAC values at 59.54 keV, the transmission percentages for the SSP0, SSP5, SSP10, SSP20, and SSP30 samples were calculated as 95.89%, 94.06%, 93.83%, 93.34%, and 92.99%, respectively, clearly demonstrating a decrease in photon transmittance with increasing SSP content.

In addition to MAC and LAC, shielding performance was further assessed using the mean free path (MFP) and half-value layer (HVL) parameters. These parameters are inversely related to a material’s ability to attenuate radiation—lower values indicate better shielding efficiency. As shown in Figure 10c,d, both MFP and HVL increase with photon energy, reflecting the decreased likelihood of photoelectric interaction at higher energies. At a photon energy of 13.37 keV, the MFP for the unfilled sample was 0.12 cm, whereas the SSP30 composite exhibited a reduced MFP of 0.062 cm. Similarly, at 51.74 keV, the MFP decreased from 3.21 cm (neat polymer) to 1.83 cm (SSP30), representing a 65% improvement in attenuation due to SSP incorporation. Notably, the MFP of natural animal bone at 59.5 keV has been reported as 6.397 cm, while the SSP30 composite in this study demonstrated a significantly lower MFP of 2.20 cm at the same energy. This indicates that SSP is more effective than natural bone in attenuating low-energy gamma radiation, emphasizing its suitability for shielding applications in biomedical or industrial fields [50].

The effective atomic number (*Z_eff_*), akin to the atomic number of pure elements, serves as a critical parameter in evaluating the interaction of composite materials with ionizing radiation. It effectively represents the overall atomic interaction potential of heterogeneous systems. Similarly to the mass attenuation coefficient (MAC), Z_eff_ is influenced by dominant photon–matter interaction mechanisms—namely, the photoelectric effect, Compton scattering, and pair production—which vary with photon energy.

In the low-energy region (<100 keV), MAC exhibits strong sensitivity to Z_eff_, following a power–law relationship roughly proportional to $Z_{eff}^{4}$ to $Z_{eff}^{5}$. As a result, even small increases in Z_eff_ can lead to substantial enhancements in attenuation performance. In the intermediate-energy range (100–1000 keV), where Compton scattering predominates, MAC becomes more dependent on electron density, and the correlation with Z_eff_ weakens. At high photon energies (>1.022 MeV), pair production becomes significant, and MAC is approximately proportional to $Z_{eff}^{2}$, restoring the importance of Z_eff_ at these energy levels.

**Figure 11:**
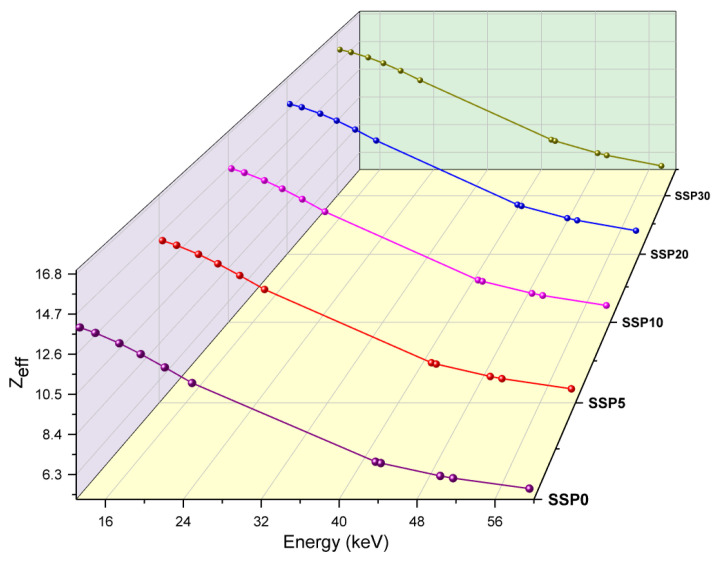
Z_eff_ values of PVC/PSt/SSP composite films.

In this study, Z_eff_ values for the PVC/PSt/SSP composite samples were experimentally evaluated across a range of low photon energies. As shown in Figure 11**,** the SSP30 sample exhibited the highest Z_eff_ values, indicating superior attenuation efficiency, while SSP5 demonstrated the lowest values across the entire energy spectrum.

The energy build-up factor (EBF) quantifies the contribution of both primary (unscattered) and secondary (scattered) photons during the interaction of gamma or X-ray photons with shielding materials. Unlike the MAC, which only accounts for the attenuation due to direct photon absorption, the EBF incorporates the effects of multiple scattering events that increase the photon fluence within the material. This distinction is especially important in scenarios involving high-energy photons or thick shielding layers, where Compton scattering significantly contributes to the total radiation dose.

**Figure 12:**
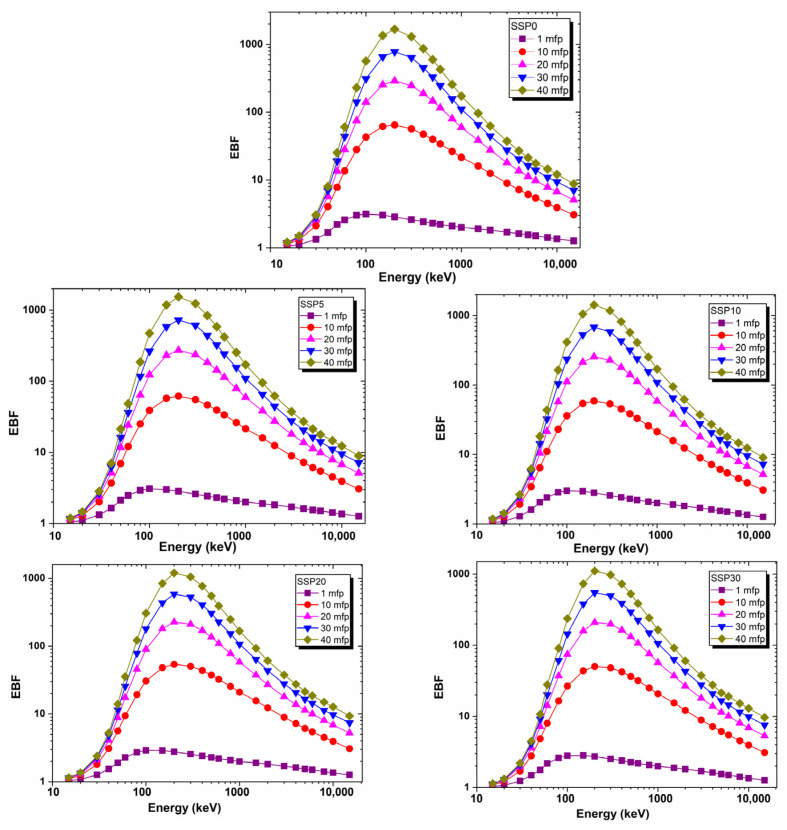
EBF values of PVC/PSt/SSP composite films.

Using the EpiXS computational model, EBF values were calculated for the composite films over a wide photon energy range from 15 keV to 15 MeV. As illustrated in Figure 12, the EBF values were lowest at low photon energies, attributed to the dominance of photoelectric absorption. In this energy range, most incident photons are absorbed before undergoing significant scattering, resulting in minimal build-up effects.

At a photon energy of 200 keV and a penetration depth of 40 mean free paths (mfp), the energy build-up factor (EBF) values were found to be 1672, 1472, 1470, 1127, and 1045 for the SSP0, SSP05, SSP10, SSP20, and SSP30 samples, respectively. These values reflect the extent of photon build-up due to multiple scattering events and align with the trends observed in the attenuation coefficients (MAC and LAC) for each composition. Generally, materials with higher absorption coefficients exhibit lower build-up factors, particularly in energy regions where Compton scattering is less dominant. Accordingly, the EBF results support the absorption-based analysis and confirm that the SSP30 sample possesses the most effective shielding capability among the studied composites.

#### 3.5.2. Albedo Number

**Figure 13:**
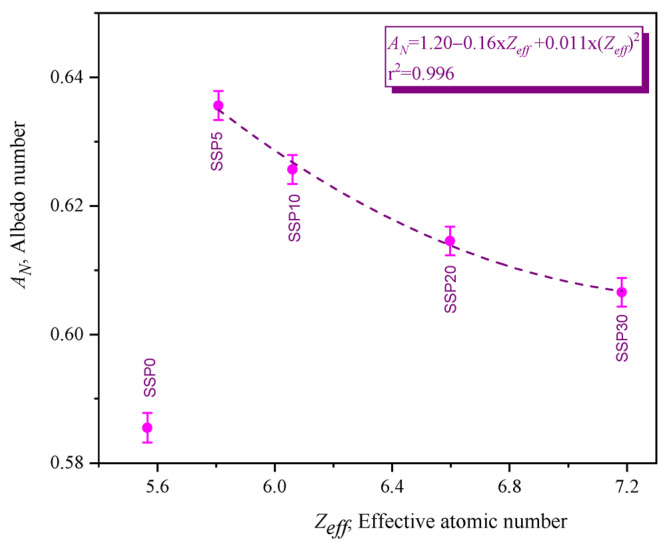
Albedo numbers of PVC/PSt composite films.

In photon–matter interactions, absorption and scattering represent complementary mechanisms: absorption results in energy deposition within the material, while scattering redirects photons, contributing to reflection. The albedo number, or anti-reflection coefficient, of the PVC/PSt/SSP composites was calculated and is presented in Figure 13.

Among the samples, SSP5 exhibited the highest reflectance, indicating the greatest scattering effect. Conversely, SSP30 showed the lowest albedo value, consistent with its enhanced absorption performance. This trend supports the data from the attenuation coefficients, suggesting that increasing SSP content—especially CaCO_3_—suppresses photon backscattering and enhances absorption.

According to the albedo number equation provided in Table 3, the albedo is mainly influenced by the energy and intensity of Compton-scattered photons. As these photons lose energy while traversing the medium, a decrease in scattering intensity results in a corresponding decline in the albedo number. Thus, increasing SSP content improves the anti-reflective behavior of the PVC/PSt matrix.

Specifically, the addition of 5% SSP to the PVC/PSt blend increased photon reflectance by approximately 8.56%. However, increasing the SSP content from 5% to 30% led to a 3.60% reduction in reflectivity, highlighting the role of CaCO_3_ in minimizing backscattering effects. Overall, the synthesized PVC/PSt-based composites—particularly those incorporating CaCO_3_—demonstrate promising properties for use as anti-reflection coatings in gamma radiation-shielding applications.

Additionally, the Z_eff_ values of the composites at 59.54 keV were computed using the EpiXS code, and their relationship with the albedo number is illustrated in Figure 13. A second-degree polynomial trend was observed between Z_eff_ and the albedo number, reinforcing the dependence of scattering behavior on the material’s effective atomic number.

#### 3.5.3. Neutron Attenuation

**Figure 14:**
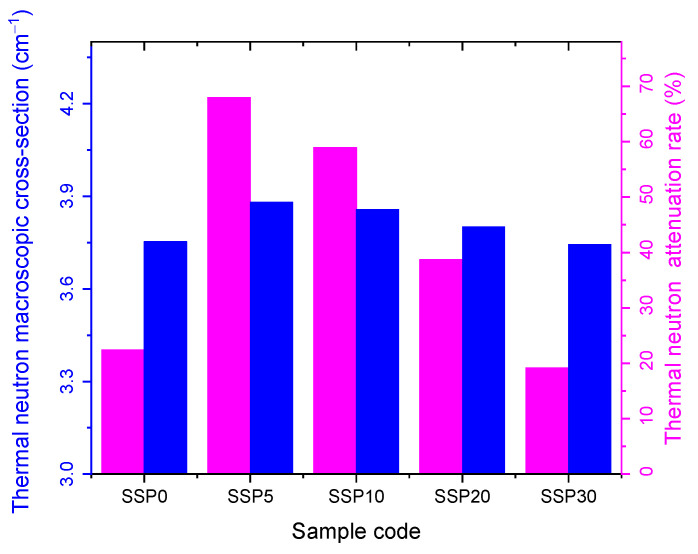
Thermal neutron macroscopic cross-section and attenuation rate graph of PVC/PSt/SSP composite films.

Figure 14 illustrates the thermal neutron macroscopic cross-section and the corresponding attenuation rate for 1.6 mm thick PVC/PSt composite films. The attenuation rate serves as an indicator of the material’s neutron shielding efficiency. Among the tested samples, SSP5 exhibited the highest attenuation performance against thermal neutrons. As the SSP content increased from 0% to 5%, the thermal neutron attenuation rate improved, rising from 16.9%. The improved neutron attenuation performance at 5% SSP (SSP5) arises from a favorable balance between the hydrogen content of the polymer matrix and the added filler’s contribution to macroscopic cross-section. SSP (i.e., CaCO_3_) contributes negligible hydrogen, so as SSP loading increases, the overall hydrogen atom density in the composite declines. Because hydrogen is exceptionally effective at moderating fast neutrons via elastic scattering (owing to its mass being close to that of a neutron) excessive substitution of polymer mass with non-hydrogenous filler reduces neutron moderation efficiency. Thus, beyond 5% SSP, the dilution of hydrogen outweighs the benefit from increased macroscopic cross-section, causing the attenuation rate to decline.

**Figure 15:**
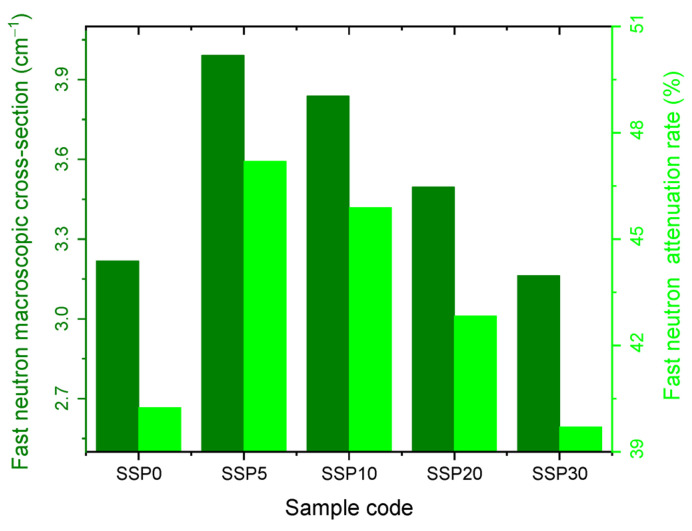
Fast neutron macroscopic cross-section and attenuation rate graph of PVC/PSt/SSP composite films.

Figure 15 presents the fast neutron macroscopic cross-section and corresponding attenuation rates. Similarly to the thermal neutron results, the SSP5 sample demonstrated the highest protective efficiency against fast neutrons, with the attenuation rate increasing from 17.3% as SSP content rose from 0% to 5%. However, beyond this point, the attenuation capacity declined, as reflected in the order of macroscopic cross-sections for both thermal and fast neutrons: SSP30 < SSP20 < SSP10 < SSP5, as shown in Figure 14 and Figure 15.

The neutron shielding parameters for all samples are summarized in Table 5. The data confirm that the SSP5 composite offers the most effective neutron shielding performance, as evidenced by its lowest mean free path (MFP) and half-value layer (HVL) values.

Additionally, neutron equivalent dose values were evaluated for the PVC/PSt/SSP composite films through a series of experimental measurements. The background dose rate was recorded at 1.0577 μSv/h. To ensure reliability, multiple measurements were taken, and outlier data were excluded based on statistical inconsistency, considering the stochastic nature of neutron interactions and detector variability.

**Figure 16:**
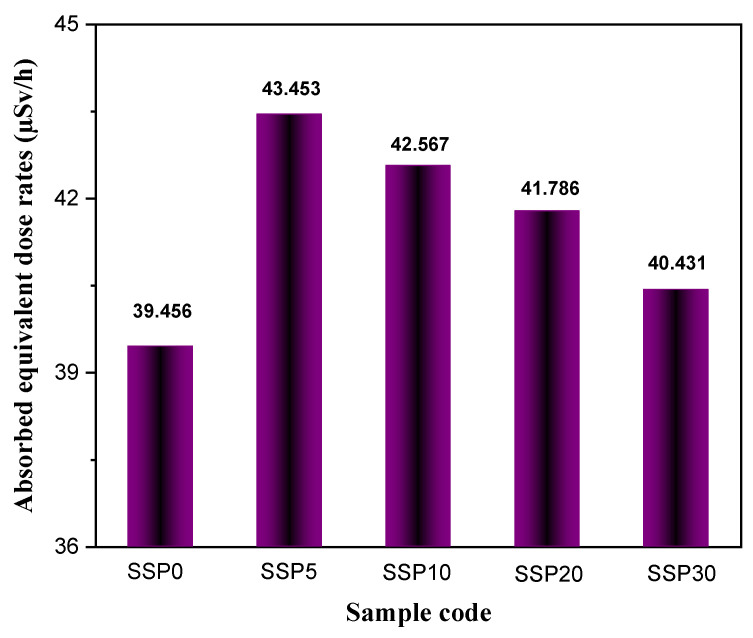
Neutron absorbed equivalent dose rates (µSv/h) of PVC/PSt composite films.

Figure 16 displays the absorbed neutron equivalent dose rates (μSv/h) for each sample. The inclusion of SSP was found to significantly enhance the neutron attenuation capability of the PVC/PSt blend. In Figure 16, the neutron dose measurements were evaluated based on multiple repeated experiments and background correction. The resulting measurement uncertainty was found to be less than 2.1%, and this has been indicated using error bars in Figure 16.

Notably, the SSP5 sample absorbed 43.45% of the dose from the fast neutron source, compared to 39.46% for the unmodified blend (SSP0). This represents an approximate 11% improvement in neutron absorption with the addition of 5% SSP. Interestingly, higher SSP concentrations beyond 5% were associated with reduced neutron absorption efficiency, likely due to compositional or structural factors limiting effective neutron interaction.

In order to better interpret the attenuation behavior of the PVC/PSt/SSP composites, we included a comparative assessment with other biogenic and waste-derived materials reported in the literature. Table 6 presents the mass attenuation coefficient (MAC), mean free path (MFP), and half-value layer (HVL) values at 59.54 keV for selected composites. Among them, the SSP30 sample demonstrates superior attenuation performance compared to natural bone and eggshell-reinforced systems, particularly in terms of MAC and MFP. Although PbO-based composites show higher attenuation, their known toxicity and environmental drawbacks restrict their applicability. On the other hand, SSPs stand out as a safer, low-cost, and sustainable alternative for photon shielding, especially in biomedical and environmental contexts.

## 4. Conclusions

In this study, the radiation-shielding capabilities of five varieties of PVC/PSt composite films were investigated and the main findings can be compiled as follows:

❖ It has been observed that snail shells have higher absorbent than animal bones in the literature.
❖ The radiation shielding and anti-reflective performance of snail shell-based polymer films suggests that powdered snail shells could also be used as additives in concrete-based materials for high-energy photon applications.
❖ C and O elements in the SSP structure affect fast and thermal neutron cross-sections.
❖ It was found that the radiation-shielding behavior followed the following order:
❖ SSP30 > SSP20 > SSP10 > SSP5 for X-rays.
❖ The order of thermal and fast neutron macroscopic cross-section is SSP30 < SSP20 < SSP10 < SSP5.
❖ The neutron absorption equivalent dose rates (μSv/h) of the composite films behavior followed the following order: SSP30 < SSP20 < SSP10 < SSP5.
❖ The film with the highest reflection efficiency is SSP5. The film with the highest anti-reflection is SSP30.
❖ The PVC/PSt/SSP composite films demonstrated significant photon and neutron attenuation capabilities, along with anti-reflective properties, indicating their potential for use in radiation-shielding applications, including those relevant to nuclear or medical environments.

To conclude, this study introduces a polymer-based composite material reinforced with SSPs as a promising candidate for low-energy photon shielding. Attenuation measurements conducted at 59.54 keV demonstrated that the material performs effectively, with experimental MAC values aligning closely with theoretical expectations. In addition to its absorption capacity, the material also exhibited measurable anti-reflective behavior, which may offer added value in applications where reflected radiation is undesirable.

While traditional shielding materials such as lead and tungsten are widely used in high-energy gamma or neutron radiation settings, they are less suitable for applications requiring lightweight, flexible, and non-toxic solutions. The SSP-based composites developed in this work were specifically designed for such conditions, especially for photon energies below 100 keV.

Beyond medical and dental imaging, low-energy radiation is widely used in other sectors. For example, low-energy X-rays (typically 40–100 keV) are employed in the food industry for microbial reduction, in pharmaceutical sterilization systems, in non-destructive testing (NDT), and in the quality control of electronic components. In these contexts, there is a growing demand for sustainable and lead-free shielding options that are both efficient and safe for users and environments. The biocompatibility and natural origin of SSP offer additional advantages in these settings.

In dental radiology in particular, the SSP-reinforced composite holds potential as an intraoral protective layer to help reduce radiation-induced damage, such as dental caries, during head-and-neck imaging or treatment.

Overall, the findings suggest that SSP-based composites—particularly SSP30—are not intended to replace heavy-metal shields in high-energy environments but rather to serve as a sustainable and functional alternative for targeted low-energy radiation shielding, where factors such as weight, flexibility, and material safety are key design considerations.

## Figures and Tables

**Table 1:** The sample codes, weight fractions (%) of compounds and densities of each PVC/PSt/SSP blends.

Sample Codes	PVC	PSt	SSP	Density (g/cm^3^)
SSP0	0.375	0.625	0	1.058
SSP5	0.353	0.588	0.059	1.381
SSP10	0.331	0.551	0.118	1.402
SSP20	0.288	0.481	0.231	1.436
SSP30	0.248	0.413	0.340	1.485

**Table 2:** X-ray emission energies of radioactive source.

Target Selected	Energy (keV)	
	K_α_	K_β_
Rb	13.37	14.97
Mo	17.44	19.63
Ag	22.10	24.99
Tb	43.74 (K_α2_) and 44.48 (K_α1_)	50.38 (K_β1_) and 51.71 (K_β2_)

**Table 3:** Equations and definitions used in the present study for evaluating radiation and neutron shielding parameters.

Parameters	Equations	Descriptions
Linear attenuation coefficients, (LAC, cm^2^/g) ^1^	$\mu =ln\left(I_{0}/I\right)/t$	$I_{0}$ and I are the intensities of the incident photons and those passing through the absorber, respectively.
Mass attenuation coefficients, (MAC, cm^2^/g) ^1^	$\mu_{m}=ln\left(I_{0}/I\right)/\rho t$	ρ is the density and t is thickness.
Mean free path, (MFP, cm) ^1^	MFP=1/μ	μ is the linear attenuation coefficient (1/cm).
Half value thickness, (HVL, cm) ^1^	HVL=0.693/μ	μ is the linear attenuation coefficient (1/cm).
Effective atomic number ^1^	$Z_{eff}=\sigma_{t,a}/\sigma_{t,e}$	The total electronic cross-section is $\sigma_{t,e}$ and the total atomic cross section is $\sigma_{t,a}$
Albedo number ^2^	$A_{N}=\left[\frac{N_{Comp}/\varepsilon {(E}_{Comp})}{N_{coh}/\varepsilon (E_{coh})(\frac{1}{d\Omega })(1/2)}\right]$	dΩ is the solid angle, $N_{Comp\;}$and $N_{coh\;}$are the areas of Compton and coherent scattered peaks. The photo-peak efficiencies are $\varepsilon {(E}_{Comp})$ and $\varepsilon (E_{coh})$ in Compton (48.87 keV) and coherent (59.54 keV) scattered energies.
Build-up factor ^1^	$I=BI_{0}e^{-\mu_{m}\rho t}$	B is build up factor.
Fast neutron removal cross-section ^3^	$\sum_{R}=\sum_{i}W_{i}{\left(\sum_{R/\rho }\right)}_{i}$	Fast neutron removal cross-section of the *i*th element is denoted by $\sum_{R/\rho }$, while $W_{i}$ represents its partial density.
Fast neutron macroscopic cross-section ^4^	$\Sigma_{R}=ln\left(I_{0}/I\right)/t$	$I_{0}$ and I are incident and transmitted intensities for fast neutrons, respectively, within the energy range of 0.8–11 MeV and t is absorber thickness.
Thermal neutron cross-section ^4^	$\Sigma_{R}=ln\left(I_{0}/I\right)/t$	$\Sigma_{R}$ is the probability of thermal neutrons (25.4 meV) being retained in a unit length of matter.
Mean free path (neutron)	$\mathrm{MFP}=1/\Sigma_{R}$	MFP is the part traveled by a neutron between two collisions.
Half value thickness (neutron)	$\mathrm{HVL}=1/\Sigma_{R}$	*HVL* is the thickness of the material that reduces incoming neutron radiation by half.
The absorbed equivalent dose percentage/Neutron attenuation rate	${(I}_{0}-I)/I_{0}\times 100$	$I_{0}\;$and I are the intensities of incident neutrons and those passing through the absorber, respectively.

^1^ The EpiXS software (2.0.1.Dark) program is used to calculate theoretical values [28]. ^2^ The calculating procedure of albedo numbers is available from our previous work [27]. ^3^ Theoretical values are calculated using the Phy-X/PSD software (v1.0) package [29]. ^4^ Theoretical values are calculated using the NGcal software (v1.0) package [30].

**Table 4:** Theoretical and experimental MAC values (cm^2^/g) of PVC/PSt/SSP samples.

Energy (keV)	SSP0		SSP5		SSP10		SSP20		SSP30	
	Theo.	Exp.	Theo.	Exp.	Theo.	Exp.	Theo.	Exp.	Theo.	Exp.
13.37	7.7908	8.1850 ± 0.3942	8.2974	8.1189 ± 0.1785	8.8040	8.6028 ± 0.2012	9.8172	10.1733 ± 0.3560	10.8305	11.3561 ± 0.6064
14.97	5.6489	5.8837 ± 0.2347	6.0169	6.3116 ± 0.2947	6.3849	6.1636 ± 0.2213	7.1209	7.3942 ± 0.2733	7.8569	8.1247 ± 0.4339
17.44	3.6727	3.7939 ± 0.1212	3.9104	4.1309 ± 0.2205	4.1480	4.3836 ± 0.2356	4.6234	4.8914 ± 0.2680	5.0988	5.3678 ± 0.2866
19.63	2.6430	2.7619 ± 0.1189	2.8120	2.8696 ± 0.0576	2.9809	3.1447 ± 0.1637	3.3189	3.4469 ± 0.1280	3.6569	3.8679 ± 0.2065
22.10	1.9137	1.8661 ± 0.0477	2.0334	2.1123 ± 0.0789	2.1530	2.0516 ± 0.1014	2.3923	2.3139 ± 0.0784	2.6316	2.7568 ± 0.1472
24.90	1.3979	1.3606 ± 0.0373	1.4822	1.5371 ± 0.0549	1.5665	1.5244 ± 0.0421	1.7350	1.6902 ± 0.0448	1.9036	1.9979 ± 0.1067
43.74	0.4043	0.4242 ± 0.0199	0.4199	0.3961 ± 0.0238	0.4355	0.4152 ± 0.0204	0.4667	0.4432 ± 0.0236	0.4980	0.5157 ± 0.0275
44.28	0.3959	0.4077 ± 0.0118	0.4110	0.4233 ± 0.0123	0.4260	0.4115 ± 0.0145	0.4560	0.4668 ± 0.0107	0.4861	0.4988 ± 0.0266
50.38	0.3242	0.3428 ± 0.0186	0.3344	0.3473 ± 0.0129	0.3444	0.3363 ± 0.0081	0.3646	0.3459 ± 0.0187	0.3848	0.3957 ± 0.0211
51.70	0.3126	0.2945 ± 0.0181	0.3219	0.3312 ± 0.0093	0.33125	0.3475 ± 0.0163	0.3498	0.3679 ± 0.0180	0.3685	0.3757 ± 0.0201
59.54	0.2622	0.2485 ± 0.0138	0.2683	0.2773 ± 0.0090	0.27426	0.2843 ± 0.0101	0.2862	0.2997 ± 0.0135	0.2982	0.3057 ± 0.0163

**Table 5:** The neutron shielding parameters of PVC/PSt composite films.

The thermal neutron attenuation parameters of PVC/PSt films (25.4 meV)					
Parameter	SSP0	SSP5	SSP10	SSP20	SSP30
∑ (cm^−1^)	3.4024	4.219	4.058	3.695	3.344
MFP (cm)	0.2939	0.2370	0.2464	0.2706	0.2990
HVL (cm)	0.2034	0.1640	0.1705	0.1873	0.2069
The fast neutron attenuation parameters of PVC/PSt films (4 MeV)					
Parameter	SSP0	SSP5	SSP10	SSP20	SSP30
∑ (cm^−1^)	3.218	3.991	3.838	3.495	3.162
MFP (cm)	0.3107	0.2506	0.2605	0.2861	0.3162
HVL (cm)	0.2150	0.1734	0.1803	0.1980	0.2188
The fast neutron removal attenuation parameters of PVC/PSt films.					
Parameter	SSP0	SSP5	SSP10	SSP20	SSP30
∑ (cm^−1^)	0.0860	0.1095	0.1064	0.1015	0.0975
MFP (cm)	11.6279	9.1324	9.3985	9.8522	10.2564
HVL (cm)	8.0465	6.3196	6.5038	6.8177	7.0974

**Table 6:** Photon-shielding parameters of SSP-reinforced and reference composites at 59.54 keV.

Composite Material	MAC (cm^2^/g)	MFP (cm)	HVL (cm)	Reference
PVC/PSt/SSP30 (This work)	0.3057	2.20	1.53	This work
Natural animal bone	0.2150	6.40	4.43	[45]
Eggshell-reinforced epoxy	0.2460	~3.80	~2.64	[19]
Snail shell HAp in epoxy	0.2940	2.40	1.65	[18]
PbO-reinforced PVC	0.5500	1.48	1.02	[22]

## Data Availability

The data that support the findings of this study are available from the corresponding author upon reasonable request.
